# Modeling the Use of Online Knowledge Community: A Perspective of Needs-Affordances-Features

**DOI:** 10.1155/2021/3496807

**Published:** 2021-12-27

**Authors:** Zeyu Jiao, Jianbin Chen, Eunjin Kim

**Affiliations:** ^1^Kyonggi University, Suwon 443-760, Gyeonggi-do, Republic of Korea; ^2^Beijing Union University, Beijing 100101, China

## Abstract

With the support of network information technology, the Online Knowledge Community (OKC) has emerged. Among different OKC applications, some entered into the new era of popular knowledge production, while others experienced the process to decline. In order to solve the dilemma faced by the OKC platforms, the needs-affordances-features (NAF) perspective on OKC is proposed by taking Zhihu, one of the most popular OKC applications in China as an example. According to NAF, the psychological needs of individuals motivate the use of Zhihu to the extent to which Zhihu offers affordances that satisfy the individuals' needs. By collecting data through questionnaires and using SPSS and AMOS for data analysis, the relationship between individuals' psychological needs and Zhihu affordances is identified. This paper generates two aspects of implications. In terms of theoretical implications, a more comprehensive framework is developed for the affordances of OKC as a whole, and the NAF model is leveraged to identify related psychological needs which motivate the use of a specific OKC application—Zhihu. Further research can leverage NAF to identify different OKC platform features which satisfy the psychological needs of their targeting users to optimize the system of OKC platforms. As for practical implications, by building the relationship between the affordances of OKC platforms and users' psychological needs, marketers have to realize that although the digital platform system has a certain degree of imitability, the value positioning, user community, and core capabilities behind those platforms are all different. Thus, the platform system must also be differentiated. In order to determine the appropriate business model, a clear understanding of these organizational features should be identified.

## 1. Introduction

Knowledge management has attracted the attention of many researchers due to its close relationship with organizational performance [1]. In the Internet age, the “Online Knowledge Community” (OKC) that integrates the functions of “knowledge sharing” and “online social networking” has emerged. With the opening of registration restrictions, a large number of ordinary users have poured in. Representative OKC platforms such as Douban and Zhihu have entered the second stage of development from the early stage of highly acclaimed and rapid development: the era of popular knowledge production [2]. The vast information capacity and random insertion and editing at any time have significantly increased the amount of information and the level of confusion, which changed the traditional online communication methods [3]. At the same time, the operation of knowledge communities is facing a complex situation. For example, although Wukong Questions and Answers (Q&A) application persuaded over about 300 influencers of Zhihu to join with a high salary in 2017, the server of Wukong Q&A ceased operation in 2021 [4]. During the same period, Zhihu ushered in its tenth birthday celebration, and there were rumors of listing on the stock market. There is the success of Zhihu, while there is also the shutdown of Encarta, the failure of Google Answers, the decline of Wikipedia [5, 6], and the quit of Wukong Q&A, which operated less than four years independently.

Of these OKC platforms, why do high-quality creators prefer Zhihu? From a social-technical perspective, the OKC platform is a collection of users' behavioral possibilities and needs in social media and organizational environments [7]. Its value is created by digital technology and is cocreated by the interaction between the user, the technology, and the purpose of use [8]. Ongus [9] also mentioned that the quality and relevance of technology resources and information content are closely related to users' needs. Therefore, the platform functions or resources enabled by platform features are essential for users' needs. However, the current literature has not yet studied this mechanism, resulting in a “black box” of the platform system. In the research of Facebook using motivations, the needs-affordances-features (NAF) model was first suggested by focusing on platform characteristics in the context of social media [10]. According to NAF, the psychological needs of individuals motivate the use of applications to the extent to which these applications offer affordances that satisfy individuals' needs (ibid). As OKC is similar to social media given the “online social networking” functions, the NAF model is proposed to address the following questions:

1. What innate psychological needs do people seek to satisfy through OKC?
2. What are the main affordances provided by OKC?
3. Which of these OKC affordances meet which psychological needs?

In this paper, according to the literature review, the needs-affordances-features (NAF) model is used to identify the relationship between psychological needs, affordances, and features. Based on the research of psychological needs, OKC affordances, and Zhihu features, hypotheses of this paper are suggested, and the conceptual framework is sorted out. In the final, from data collection and analysis, the relationship between individuals' psychological needs and Zhihu affordances is identified. The result shows that each of the Zhihu affordances is likely related to fulfilling some psychological needs. For example, the need for autonomy can motivate people to use Zhihu that has the affordance of browsing others' content, while the need for relatedness can motivate the use of Zhihu that has affordances of self-presentation, relationship formation, and meta-voicing.

In terms of the research gap, although current research has noticed that the platform's community ecology and value cocreation may play an essential role in optimizing the platform, the platform is mainly regarded as an intermediary connection point and ecological core node [11, 12]. That is to say, there is a lack of analysis of the internal micromechanism based on the platform's individualization and differentiation. Therefore, this paper will address the “black box” of the platform system. With the consideration of platform affordances, the interaction relationship between the platform and users will be studied from the dimensions of core functions and resources to enrich the theoretical system of the platform further and achieve theoretical innovation. In the view of practice, from the fact that Wukong Q&A has invested billions in the construction of the community, although it still fell to the ground, it can be seen that different platform affordances could create different community ecology. The research of this topic can guide more platforms to design more effective OKC applications under the premise of unifying user needs and organizational characteristics, which could lead to avoiding waste of social resources and improving the success rate of Internet innovation.

## 2. Literature Review

### 2.1. Online Knowledge Community (OKC)

Online Knowledge Community (OKC) refers to a virtual space where users scattered in different regions carry out knowledge exchange and interpersonal interaction with the support of network information technology [13], which is a typical social-technical system [14]. OKC has the collaborative ordering of the social and knowledge systems, and users are the driving force of ordering the two systems [3, 15].

Currently, OKC research focuses on three hot areas: user research, content research, and community research [2]. User research mainly explores OKC users' behavior characteristics and patterns and analyzes user motivation for participation and the interaction between users. Content research focuses on content quality measurement and evaluation, semantic analysis, and topic recognition, influencing factor analysis, relationship research, and so forth. Community research is about conducting research on community operation mechanisms, content generation models, and community comparisons. The representative viewpoints are as follows: the field of information management emphasizes the impact of IT technology on knowledge proliferation [16]; the field of sociology pays more attention to the impact of trust on online interaction [17]. Guan et al. [18] believed that knowledge inflow is the key and examine the antecedents of knowledge contribution behavior in multiple dimensions. In contrast, Lai et al. [19] and Zhao and Huang [20] believed that knowledge search is equally important. They examine the antecedents of knowledge search behavior from the perspectives of behavior attitude, subjective norms, and ability beliefs. In terms of user research, opinion leaders or leading users usually have more experience or mastered more professional knowledge, so their behaviors are more exploratory or creative, and they are easier to gain trust. In addition, knowledge contribution is their important active behavior [21].

### 2.2. Affordance Theory

Affordance originally refers to the support that objective things can provide for a certain behavior, that is, action possibilities permitted by properties of objects [22]. In recent years, it has become more popular in organizational research and can be used to better understand the impact of the combination of new technologies and organizational characteristics on organizational innovation and operations [23]. “Affordance” provides not only a strong theoretical perspective for studying the relationship between technology and personnel in an organization but also a better language for the structured and patterned description of specific practices [24].

As a commonly used communication method, social media affordances have an important influence on the organization's communication process, employee and user behavior, and psychology, so it is currently a hot topic of affordance research. Postigo [25] first analyzed, from the perspective of social-technical interaction, how YouTube guides users to conduct behaviors that are beneficial to the commercial interests of the platform through the design of platform architecture. Rice et al. [7] defined social media affordances as the relationship between the action possibility and the need (or purpose) perceived by users aggregated in social media and the organizational environment under the constraints of the potential features/functions of the social media platforms. Majchrzak and Faraj [23] studied the relationship between the different ways through which employees participate in publicly visible conversations on social media platforms and knowledge sharing. Sun et al. [26] divided the use of social media platforms into three categories: interactivity (conversations between users and comments on related information), accessibility (users can obtain relevant information), and sociality (users establish connections with other users).

There are many research results around the dimension of affordances. Treem and Leonardi [27] proposed four dimensions of social media affordances, visibility, associating, editability, and persistence, which have been commonly used [24, 26, 28]. Majchrzak and Faraj [23] proposed four dimensions of affordances for knowledge sharing (meta voicing, trigger attending, network-informed associating, generative role-taking). In addition, there are six dimensions of functional media affordances [7], ten dimensions of communication affordances [29], and six dimensions of social business technology affordances [30]. As for new media affordances, Pan and Liu [31] proposed production affordances (including editability, reviewability, replicability, scalability, and associability), social affordances (including greet-ability, emotion-ability, coordinate-ability, and connect-ability), and mobile affordances (including portability, availability, locatability, and multimediality), which reflect the initiative of the information prosumer.

The most integrated literature is proposed by Karahanna et al. [10], who divide social media affordances into egocentric and allocentric affordances based on the theory of self-determination and psychological ownership. In addition, the research framework for social media adoption and use is proposed, which is the needs-affordances-features (NAF) model, and an empirical study of affordances is conducted with Facebook as an example. Zhang and Huang [32] discussed the realization of platform affordances from aspects of technology affordances and social affordances and emphasized that users' perceptions and actions must be considered when gaining insight into the complex relationship between technology and society.

Based on the above literature, OKC affordances could be divided into platform affordances and product affordances in this research. Platform affordances include technology affordances and social affordances. However, this paper focuses on the relationship between individuals' psychological motivations and OKC affordances. Compared to technology affordances which provide technical support for platform affordances, social affordances can better reflect the relationship between platform affordances and people's psychological needs for using a certain OKC platform. As a result, social affordances will be used as platform affordances in this research, which is realized by platform functions, such as Zhihu platform features. Product affordances focus on the affordances of OKC knowledge resources, referred to as knowledge affordances (see Table 1).

### 2.3. NAF Perspective for OKC

**Figure 1:**
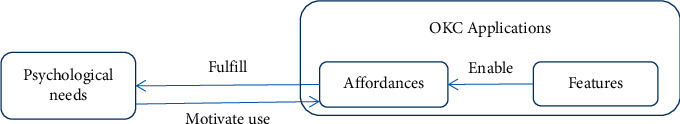
Logic of the NAF perspective.

The psychology literature shows that people are driven to engage in activities that fulfill their innate psychological needs. Therefore these psychological needs are stimulating states that act as motivations for action [33]. Based on this premise, a needs-affordances-features (NAF) perspective shows that innate psychological needs motivate people to use OKC apps, which have affordances to satisfy their psychological needs potentially. For example, OKC apps provide the affordance to connect with others, through features such as “Following” and “Chatting” on Zhihu. This affordance can be used to satisfy people's psychological needs for relatedness. Therefore, this psychological need drives them to participate in using Zhihu features that provide this affordance to meet this psychological need (see Figure 1).

### 2.4. Relationship between Psychological Needs and OKC Affordances

Although there is no research to categorize psychological needs in the OKC context, research on human motivations shows that “motivation can be conceived as a duality” [34]. On the one hand, people focus on the self. On the other hand, they strive for relationships between the self and others. Hermans [35] suggested the former motivation as the S-motive and the latter as the O-motive. Based on self-determination theory (SDT) [33, 36] and psychological ownership theory (POT) [37–39], seven psychological needs are listed, which span the two polarities and illustrate people's self-focused and other-focused motivations. Thus, these two theories are synthesized in this paper to show psychological needs in a more comprehensive way. According to the terminology of Hermans [35], these seven psychological needs are divided into the self-focused group, also called S-needs (need for autonomy, competence, having a place, coming to know the self, maintaining continuity of self-identity, and one aspect of expressing self-identity), and the other-focused group, also called O-needs (need for relatedness and one aspect of expressing self-identity).

According to Karahanna et al. [10], there are 12 social media affordances, which constitute egocentric affordances (self-presentation, content sharing, and interactivity) and allocentric affordances (presence signaling, relationship formation, group management, browsing other's content, meta-voicing, communication, collaboration, competition, and sourcing). As OKC is a typical social-technical system equipped with the function of “online social networking,” these 12 social media affordances will be used to analyze the OKC social affordances. However, in addition to social affordances, OKC has its specific product, which is knowledge. According to Shi et al. [40], knowledge affordances are defined as unique attributes of knowledge that meet the needs of consumers, which can be measured in 4 dimensions (reliability, economies, selectivity, and uniqueness). In order to map the OKC affordances to psychological needs, Table 2 is shown based on former research [10] and six OKC researchers (see Table 3 for details of term definitions).

### 2.5. Literature Gaps

First, there is a lack of platform features in the context of OKC research. At present, the OKC research has three hot areas: users, content, and communities/platforms, and OKC affordances have also attracted much attention. However, the OKC research focuses on the field of subrole characteristics and its socialization mechanism and lacks attention to platform differences. Second, people's attention to the role of psychological needs in motivating the use of the OKC platforms is limited. In particular, there has not been a comprehensive theoretical-based attempt to determine a set of psychological needs that are salient in the OKC environment. In most studies, the focus is not on theorizing around psychological needs, but the needs variable is one of the many variables included in the research model. Third, current studies did not adopt a systematic method to identify the OKC affordances that meet these needs. On the contrary, most people measure need satisfaction to confirm whether affordances meet people's psychological needs. The focus on the relationship between psychological needs and OKC affordances provides valuable operational design guidelines for information system researchers and practitioners. Our research aims to address these gaps.

## 3. Research Model and Hypotheses

There are three steps of developing the NAF model in the use of the Zhihu application. Firstly, to understand what psychological motivations of using this app, the affordances provided by the Zhihu platform should be identified. In 2011, the mission of Zhihu was officially launched, which is “allowing people to share knowledge, experience and insights better and find their own answers” [58]. After nearly ten years of development, Zhihu announced the brand renewal and upgrade at the 2021 New Knowledge Youth Conference. The brand slogan has been upgraded from “If there is a question, go to Zhihu” to “If there is a question, there will be an answer” (ibid). These show that self-presentation, content sharing, browsing others' content, and sourcing are salient affordances of Zhihu. Based on the functions of chatting and live Q&A, liking or collecting others' content, and voting for what others posted, it is obvious that communication and meta-voicing are also affordances of Zhihu. In addition, there is a function of “Quanzi” in Zhihu, which offers a community for the group of people who have common interests, where they could share and communicate about their experience and opinions in their own circle. As a result, relationship formation is also one of the social affordances of Zhihu. After identifying salient affordances, the most popular features of Zhihu were selected from a synthesis of research on the condition that affordances are provided by specific features of an object [52, 57, 59, 60]. With the combination of its knowledge properties, six researchers with research expertise in OKC (three faculty and three doctoral students) are asked to map the features to the 13 OKC affordances listed in Table 2.  Table 4 illustrates the result of the mapping of Zhihu features to Zhihu affordances. Therefore, Zhihu affordances are identified as self-presentation, content sharing, relationship formation, browsing others' content, meta-voicing, communication, sourcing, and knowledge attributes.

In order to predict which Zhihu affordances meet which psychological needs, the hypotheses are suggested as follows.

According to Deci [61], autonomy is defined as people's innate psychological needs to act authentically in their own life. Everyone is inherently eager for autonomy [62]. They participate in activities, not because of social norms or pressures but because they are free to choose [33, 63]. Therefore, we suggest that self-presentation, content sharing, relationship formation, browsing others' content, and sourcing are affordances that motivate users to use Zhihu. For example, those affordances allow individuals of Zhihu to choose how to present themselves (through uploading video and photos, disclosing locations), what content to share, whom to follow, what kind of community to join, and what questions to suggest or answer, which leads to hypothesis 1.

  H1: The need for autonomy can motivate the use of Zhihu that has these affordances: self-presentation, content sharing, relationship formation, browsing others' content, and sourcing.
  In 1991, Deci and Ryan illustrated that relatedness is individuals' innate psychological need to be connected to others. In 2000, Deci and Ryan extended the definition as the need “to love and care and to be loved and cared for.” Thus, we posit that a set of Zhihu affordances—self-presentation, relationship formation, browsing others' content, meta-voicing, and communication—can help people satisfy the need for relatedness by creating social connections with others. This can be realized by reaching a lot of users, joining an online group, knowing what others are doing, reacting to others' posts, and so forth [64, 65]. For example, in the context of Zhihu, users can increase social interactions by posting personal information (self-presentation), following a user (relationship formation), browsing others' posts (browsing others' content), voting for a post (meta-voicing), sending an instant message to others (communication), and so forth. Therefore, the H2 is proposed as follows.
  H2: The need for relatedness can motivate the use of Zhihu that has these affordances: self-presentation, relationship formation, browsing others' content, meta-voicing, and communication.
  Bauer and McAdams [66] analyzed that competence refers to people's innate psychological needs to deal with their environment effectively. It is the psychological needs that can be achieved by having an impact on individuals or the environment. White [67] showed that individuals need to feel competent in controlling or altering the environment and finding opportunities to increase their own knowledge or skills. Therefore, people who have a high demand for competence are more likely to seek affordances that provide them with opportunities to apply or expand their knowledge in the environment. Thus, we suggest that the affordances of meta-voicing, sourcing, and knowledge attributes can fulfill individuals' needs for competence by providing feedback to others' posts, responding to others' questions, searching answers, subscribing to valuable content, attending online courses, and so on. Thus, hypothesis 3 is suggested.
  H3: The need for competence can motivate the use of Zhihu that has the following affordances: meta-voicing, sourcing, and knowledge attributes.
  In terms of having a place, it refers to people's innate psychological needs to possess a place where they can create their own space [68, 69]. Pierce et al. [70] suggest that a sense of having a place can be partly realized by the personalization of individuals' surroundings. Applying this definition to cyberspace, users can fulfill their needs for having a place by investing time, energy, or emotion in creating their own virtual world through self-presentation (such as posting personal photos or videos on Zhihu) and content sharing (such as sharing others' content on Zhihu). In this way, a sense of having a place can be created by engaging in personalizing the cyberspace. As a result, hypothesis 4 can be proposed.
  H4: The need for having a place can motivate the use of Zhihu that has these affordances: self-presentation and content sharing.
  As for coming to know the self, Pierce et al. [71] illustrated it as individuals' innate psychological needs to identify who they are and learn about themselves. Self-identity can be developed through self-awareness, such as comparing themselves with others when people interact with their surroundings [72]. In addition, the sense of coming to know the self can be achieved by receiving feedback from others and seeing how other people think of themselves [73]. Thus, people can reflect their feelings, thoughts, and behaviors, which enable them to discover what kind of people they are. Therefore, the psychological needs for coming to know the self can be fulfilled by these affordances: browsing others' content (which helps individuals compare themselves to others) and meta-voicing (which enables individuals to see reflected appraisal). Therefore, hypothesis 5 is suggested.
  H5: The need for coming to know the self can motivate the use of Zhihu that has these affordances: browsing others' content and meta-voicing.
  Expressing self-identity is defined as people's innate psychological needs to communicate their identities with others [71]. For example, Zhihu users can satisfy their needs for expressing self-identity by self-presentation (through disclosing their personal information such as profile photos and education), content sharing (through sharing articles), relationship formation (through following other Zhihu users or joining online communities), meta-voicing (through commenting on or voting for others' posts), and communication (through chatting directly with other Zhihu users). Therefore, hypothesis 6 can be proposed.
  H6: The need for expressing self-identity can motivate the use of Zhihu that has these affordances: self-presentation, content sharing, relationship formation, meta-voicing, and communication.
  The final psychological needs suggested in this paper are maintaining continuity of self-identity, which shows individuals' innate psychological needs to maintain emotional connections between past and present [71]. Taking Zhihu as an example, self-presenting and content sharing enable users to see who they were and who they are by the questions they asked or answered, articles they shared, photos and videos they posted, and so forth. All the content constitutes one's self-identity. Thus, we posit hypothesis 7.
  H7: The need for maintaining continuity of self-identity can motivate the use of Zhihu that has these affordances: self-presentation and content sharing.

**Figure 2:**
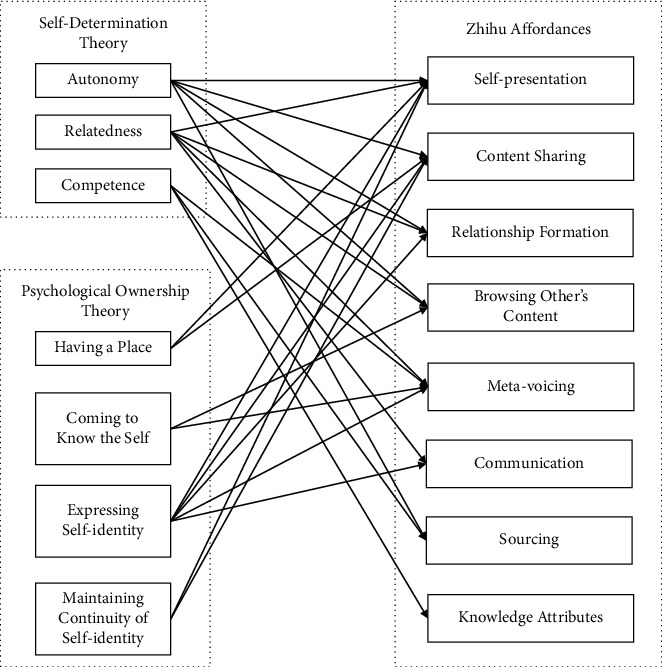
NAF model for Zhihu affordances use.

Based on the above hypotheses, the conceptual framework can be shown in Figure 2.

## 4. Research Methods

### 4.1. Questionnaire Design

Based on previous research of psychological needs and Zhihu features, questionnaire 1 and questionnaire 2 are designed to test the model of this paper. In order to measure seven variables of psychological needs, 20 question items are suggested. A adopts three-question items from Deci [61] and Deci and Ryan [33, 62, 63]. R is measured by the scale of the research from Deci and Ryan [63, 74], Jenkins-Guarnieri et al. [64], and Seder & Oishi [65]. C adopts the scale from Bauer and McAdams [66] and White [67]. HP adopts two-question items from Barki et al. [68], Harrison and Barthel [69], and Pierce et al. [70]. CK is measured by the scale of Pierce et al. [71], Festinger [72], and Mead [73] while ES and MC adopt the source from Pierce et al. [71] to be the measurement scale (see in questionnaire 1). In questionnaire 2, 17 question items are suggested to measure the extent to which the use of or feeling Zhihu features. In this study, every measurement item is measured using the five-point Likert scale. 1 for “strongly disagree,” 2 for “disagree,” 3 for “undecided,” 4 for “agree,” and 5 for “strongly agree.”

### 4.2. Data Collection

Taking into account the time constraints and sample size, the research scope is mainly selected in the first-tier cities in China that have a large number of people using Zhihu. First, the questionnaire was distributed and collected in a small range through the social network of teachers and classmates to conduct a preliminary test of the questionnaire. After the questionnaire was revised, a professional survey company was commissioned to invite Zhihu users in China's first-tier cities to respond online. The survey period was from June 2021 to July 2021. A total of 300 questionnaires were returned. After excluding uncompleted and regular answers, 300 questionnaires were finally returned, with 208 valid questionnaires, and the effective response rate was 69.3%. Based on the final sample, this paper mainly adopts SPSS26.0 and Amos24.0 software to analyze the sample data as shown in Table 5.

## 5. Results and Analysis

In this study, SPSS26.0 software and Amos24.0 software are used to analyze the research samples. Among them, the analysis methods involved in SPSS26.0 include reliability test and regression analysis, while Amos24.0 is used for confirmatory factor analysis.

### 5.1. Reliability and Validity Test

Reliability refers to the consistency or stability of the measurement results obtained by measurement tools [75]. In this paper, Cronbach's Alpha reliability test is used for testing variables. In terms of validity, it refers to the degree to which measurement tools can accurately measure the things that need to be measured [76]. This paper uses confirmatory factor analysis to test the validity of variables, which is mainly used to verify convergent validity and discriminant validity. According to Hair [76], the absolute value of factor loading should be at least 0.5 or more, and the best index value should be more than 0.7. In addition, the average variance extraction (AVE) index value should be more than 0.5. The value of the construct reliability should be higher than 0.7 to judge whether it has convergent validity. Fornell and Larcker [77] pointed out that the existence of discriminant validity should be judged based on whether the square root of AVE is higher than the correlation coefficient value between the two-factor constructs. This study uses the maximum likelihood method to estimate the model, and *χ*^2^/df, RMSEA, SRMR, NFI, CFI, TLI, IFI indicators are used to verify the model fitting degree in this paper.

It can be seen from Table 6 that the reliability of A, *R*, C, HP, CK, ES, and MC is 0.886, 0.748, 0.843, 0.767, 0.855, 0.801, and 0.832, respectively, indicating that the questionnaire has good reliability. In addition, as the result shows that *χ*^2^/df = 1.330, RMSEA = 0.040, SRMR = 0.043, NFI = 0.905, CFI = 0.974, TLI = 0.967, IFI = 0.975, it indicates that the confirmatory factor analysis model fits well. The composite reliability values of A, R, C, HP, CK, ES, and MC are 0.887, 0.752, 0.843, 0.767, 0.857, 0.808, and 0.835, which are all above 0.7, and the AVE values are 0.723, 0.503, 0.644, 0.666, 0.584, and 0.629, respectively, which are above 0.5, so it shows that the questionnaire has good convergent validity.

Table 7 shows the mean and standard deviation of A, R, C, HP, CK, ES, and MC. The AVE values of A, R, C, HP, CK, ES, and MC are 0.850, 0.709, 0.802, 0.789, 0.816, 0.764, and 0.793, respectively, which are higher than their corresponding correlation coefficients, which shows that the questionnaire has good discriminant validity.

### 5.2. Regression Analysis

Regression analysis is a statistical analysis method to determine the interdependent relationship between two variables or multiple variables. Therefore, this paper chooses regression analysis to test the hypothesis, in which gender, age, and Internet experience (in years) are used as control variables.

Table 8 shows the predictive effect of the predictor variables on the dependent variables and the magnitude of the explanation rate. Thus, the results are shown below.

Taking self-presentation as the dependent variable, it can be seen that relatedness, having a place, and expressing self-identity have a significant positive predictive effect on self-presentation (*β* = 0.223, *p* < 0.01; *β* = 0.300, *p* < 0.001; *β* = 0.219, *p* < 0.01, respectively). In addition, the explanatory rate of the predictor variables to self-presentation is 29.4%.

Taking content sharing as the dependent variable, it is shown that having a place and expressing self-identity have a significant positive predictive effect on content sharing (*β* = 0.260, *p* < 0.001; *β* = 0.179, *p* < 0.05, respectively). The explanatory rate of the predictor variables to content sharing is 15.1%.

Taking relationship formation as the dependent variable, it can be seen that relatedness and expressing self-identity have a significant positive predictive effect on relationship formation (*β* = 0.245, *p* < 0.01; *β* = 0.340, *p* < 0.001, respectively). The explanatory rate of the predictor variable to relationship formation is 25.2%.

Taking browsing others' content as the dependent variable, it is shown that autonomy and coming to know the self have a significant positive predictive effect on browsing others' content (*β* = 0.309, *p* < 0.001; *β* = 0.246, *p* < 0.01, respectively). The explanatory rate of the predictor variable to browsing others' content is 16.9%.

Taking meta-voicing as the dependent variable, it can be seen that relatedness, competence, coming to know the self, and expressing self-identity have a significant positive predictive effect on meta-voicing (*β* = 0.227, *p* < 0.01; *β* = 0.270, *p* < 0.001; *β* = 0.189, *p* < 0.01; *β* = 0.167, *p* < 0.05, respectively). The explanatory rate of predictor variables for meta-voicing is 39.7%.

Taking communication as the dependent variable, it can be seen that the predictive effects of the various variables of psychological needs are not significant.

Taking sourcing as the dependent variable, it can be seen that competence has a significant positive predictive effect on sourcing (*β* = 0.366, *p* < 0.001), and the explanatory rate of the predictor variable to sourcing is 14.9%.

Taking knowledge attributes as the dependent variable, it can be shown that competence has a significant positive predictive effect on knowledge attributes (*β* = 0.369, *p* < 0.001), and the explanatory rate of the predictor variable to knowledge attributes is 14.4%.

## 6. Discussion

From the data results above, the following observations could be reached. One is that each of the Zhihu affordances is likely to be related to fulfilling some psychological needs. At the same time, the salient psychological needs that drive people to use Zhihu could be identified. The other is that links between individuals' psychological needs and Zhihu affordances could be identified. Details are as follows.

The need for autonomy can motivate people to use Zhihu that has the affordance of browsing others' content. However, it has no relationship with self-presentation, content sharing, relationship formation, and sourcing. This means that although Zhihu users do have the freedom to determine what content to browse and when, there are some restrictions that limit users' freedom to show themselves, share content, and establish relationships. In the discussion area of the Zhihu platform, we can see many people complaining about the privacy problems of Zhihu. Some people think that it is strongly required that Zhihu set a function that can determine whether users are willing to disclose their dynamics, including the behavior of liking, collecting, and following. Otherwise, when users' friends or other people who follow the users click into the accounts, they can easily know what the users are doing or thinking in recent time. Mainly when subscribing to sensitive and private topics, users feel that their privacy has been violated. In addition, although the Internet search index improves the accuracy of predicting users' preferences and behavior [78], the recommender system used in the application may also disclose users' privacy [79]. In this case, due to social norms and impression management concerns, users may feel that their behavior cannot be entirely determined by themselves and finally think that the platform cannot meet their needs for autonomy. This view is further confirmed by the comparative study between Facebook and Twitter. Facebook's real name policy makes many users feel limited in expressing themselves, while Twitter's anonymity system reduces users' social pressure and enables people to express themselves more freely [10, 80, 81].

The need for relatedness can motivate the use of Zhihu that has affordances of self-presentation, relationship formation, and meta-voicing, but there is no significant link from relatedness to browsing others' content and communication. Although browsing others' content and communication are also likely to help users develop their relationships with others, our results show that these affordances are not able to meet the psychological needs of relatedness. This may be because when users are provided with various affordances that can meet the same psychological needs, they will choose the one that can directly meet their psychological needs. In addition, compared to social platforms such as Wechat, OKC platforms such as Zhihu have weaker functions in chatting, which may also be why people are less likely to use chatting on Zhihu to meet needs for relatedness. According to results, the features which enable affordances of self-presentation, relationship formation, and meta-voicing are used to meet needs for relatedness on Zhihu.

The need for competence is significantly related to affordances of meta-voicing, sourcing, and knowledge attributes. As a successful OKC platform, the functions provided by Zhihu, such as commenting on posts, collecting and liking, voting for posts, suggesting questions, and searching answers, as well as the high-quality content of Zhihu, meet people's demands for applying and expanding their knowledge. The emergence of OKC platforms has changed the way people obtain information. Every online interaction or behavior of searching is the process of ingesting content through the Internet. This method has brought many positive effects, such as a variety of content and quick access to information, but there will also be some negative effects, for example, how to filter meaningful information which indeed enables users to acquire competence. In order to achieve substantial development, Zhihu should avoid information redundancy and maintain high-quality content to win everyone's favor in the era of information overload.

The result supports the link between the need for having a place and the use of self-presentation and content sharing affordances. This shows that people create their own space by posting self-related content, writing the column, or sharing others' posts. Zhihu heavy users may invest a lot of time or money in engaging in Zhihu activities. In this way, people are able to be immersed in the environment they personalize for their own. As a result, the need for having a place can motivate the use of Zhihu that has affordances of self-presentation and content sharing.

The need for coming to know the self can also drive people to use Zhihu that has affordances of browsing others' content and meta-voicing. People can establish self-identity by receiving feedback from others and comparing themselves with other people [72, 73]. On the one hand, Zhihu can establish an effective feedback mechanism through such functions as liking and commenting so that users can constantly improve their answers, obtain new ideas, and may find the direction they are interested in and explore their potential. On the other hand, users will compare with their own articles or ideas by browsing other people's articles or opinions. Thus, they can find their own shortcomings and enhance their self-awareness. For example, by communicating with other users in the comment area, users may continue to be inspired and feel the differences between themselves and others to form a clearer understanding of themselves.

The result shows that individuals who have a great need to express self-identity may use Zhihu that provides affordances of self-presentation, content sharing, relationship formation, and meta-voicing. However, the affordance of communication could not fulfill people's psychological need for expressing self-identity. By joining an online community or following others (enable the affordance of relationship formation) on Zhihu, individuals are easy to express self-identity by establishing connections with other people. However, when users are high on the need for communicating their identities, the function of chatting may not be as rich as uploading their content, sharing others' content, commenting, or voting for others' posts (enable the self-presentation, content sharing, and meta-voicing affordances). In addition, as we mentioned before, compared to Wechat in China, the chatting function of Zhihu is not its superior function. Therefore, individuals are more likely to use self-presentation, content sharing, relationship formation, and meta-voicing to express self-identity.

Finally, the need for maintaining continuity of self-identity plays a nonsignificant role in driving Zhihu use.

We expect that the realization of this psychological need may only be a by-product of using Zhihu. Individuals could express their identity by posting their own content or sharing others' content. These constitute the user's previous and current experiences and records. Unless this material is specifically deleted, the information will be persistent, which provides continuity in time and a retrospective perspective for self-identity.

## 7. Implications and Future Research

### 7.1. Theoretical Implications

The characteristics of a particular OKC application provide insights into the affordances provided by that application. In addition, from the logic of NAF, this paper provides the mapping of the psychological needs that motivate using the application to its affordances. Therefore, people can predict their psychological needs based on the features provided by the OKC application. On the contrary, based on the people's level of psychological needs, it is possible to predict which affordances of the OKC application they may use.

Taking Zhihu as an example, this paper determines the psychological needs that drive the use of Zhihu from the perspective of the NAF model. Through the features of Zhihu, Zhihu affordances are identified, so the connection between affordances and psychological needs is established. Through the data collection and analysis, the results show that the salient psychological needs that motivate users to use Zhihu are autonomy, relatedness, competence, having a place, coming to know the self, and expressing self-identity, and these psychological needs are fulfilled by the affordances of browsing others' content (for autonomy), self-presentation, relationship formation and meta-voicing (for relatedness), meta-voicing, sourcing and knowledge attributes (for competence), self-presentation and content sharing (for having a place), browsing others' content and meta-voicing (for coming to know the self), and self-presentation, content sharing, relationship formation, and meta-voicing (for expressing self-identity). These results demonstrate how to use the NAF model to determine the relationship between a specific OKC platform and psychological needs.

### 7.2. Practical Implications

From the fact that Wukong Q&A has invested billions in the construction of the community although it still fell to the ground, different platform affordances could create different community ecology. The research of this topic can guide more platforms to design more effective OKC applications under the premise of unifying user needs and organizational characteristics, which could lead to avoiding waste of social resources and improving the success rate of Internet innovation. Outwardly, the digital platform system has a certain degree of imitability and reproducibility. In fact, it contains differences in corporate value pursuit, user community, and core capabilities. The platform system is a collection of affordances generated during the interaction between the organizational characteristics of the platform enterprise and the needs of users. The platform must clearly understand the dominance and decisive role of these organizational characteristics in operations. Different organizational characteristics naturally require different digital technologies and combination options to achieve differentiated functions, interfaces, and processes and produce different user interaction effects. In addition to the social affordances provided by the platform, content platforms such as Zhihu should also focus on the affordances of knowledge content, that is, whether the content resources meet the platform positioning and user needs. Furthermore, platform strategies should be used to guide content production behavior and user consumption behavior to build a good user-content collaborative ecosystem.

### 7.3. Future Research

NAF model can be extended to design science research. For example, our results can be used to provide guidance on how to develop effective OKC application features. These features should be able to meet the natural psychological needs of users. In addition, although we have studied how each type of affordance meets specific psychological needs, it is also possible that affordances have joint complementary effects. Therefore, future research can explore whether a specific combination of affordances can provide better means to meet the psychological needs or whether a specific combination of characteristics can provide specific affordances in a better way. Third, it is unclear whether OKC applications should strive to provide multiple functions to meet a single demand or provide multiple functions to meet multiple needs in order to succeed. Our empirical research in the context of Zhihu shows that although Zhihu provides a variety of affordances to meet the needs for relatedness, only three of them may be significantly related to the need for relatedness. Although other affordances may also help individuals develop relationships with others, our results seem to indicate that this is not why users use them. This may be because when individuals are provided with multiple affordances that can meet the same psychological needs, they will choose the affordance that most directly meets the psychological needs. This is worthy of further study. Finally, we mentioned earlier that compared to other Q&A platforms, Zhihu has carried out in-depth content production and provided online and offline knowledge products. Whether users meet their psychological needs online or offline may depend on whether OKC provides better functions to meet their needs than offline. These questions are interesting directions for future design scientific research and provide potential ways for future OKC research to reveal how to attract users effectively.

## Figures and Tables

**Table 1:** OKC affordances concept system.

Affordance concept system			Platform factors
OKC affordances	Platform affordances	Social affordances	Platform functions
	Product affordances	Knowledge affordances	Platform resources

**Table 2:** Mapping of the psychological needs to OKC affordances.

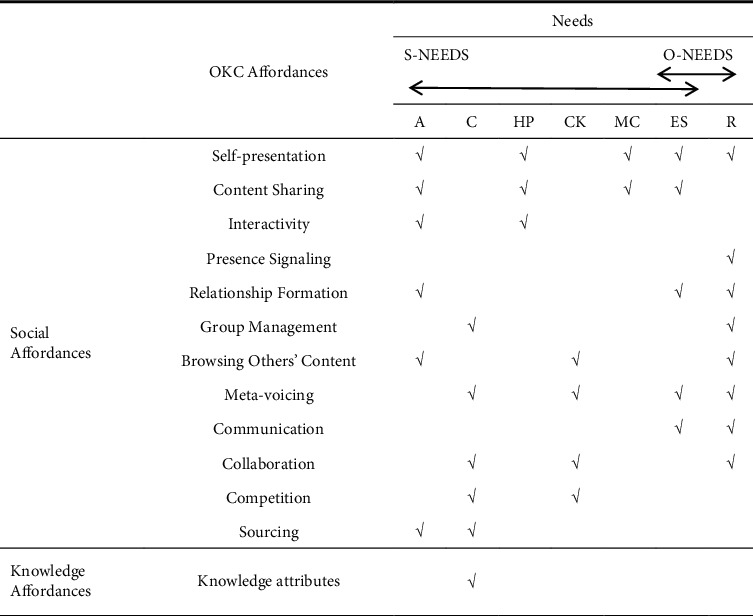

Note: A = autonomy; C = competence; HP = having a place; CK = coming to know the self; MC = maintaining continuity of self-identity; ES = expressing self-identity; R = relatedness.

**Table 3:** OKC affordances.

Affordances	Definitions	Example features
Social affordances		
Self-presentation	Users can display and present information related to themselves. This includes sharing information that somehow portrays users and shows who they are, their values and preferences, their expertise, etc. Updating descriptive information about themselves, such as gender, occupation, and location; and posting content involving pictures and videos related to themselves [41–44]	Posting my own content on Zhihu; sharing my own video on YouTube; updating my profile on Zhihu; writing personal opinions on Zhihu
Content sharing	Users can share and distribute content unrelated to them to others (e.g., sharing posts, video) [27, 45, 46]	Sharing links of other people's articles on Zhihu; sharing others' videos and photos on Instagram
Interactivity	Users can walk around in real time and change their virtual environment (e.g., to build in-world artifacts) [41]	Moving around in World of Warcraft
Presence signaling	Users can indicate their existence and know whether other users are accessible [43, 44, 47]	“Who is online” on Instagram
Relationship formation	Users can establish relationships with others, including joining groups or online communities [27, 45]	Following other users on Zhihu; joining an online community (e.g., “Quanzi” on Zhihu)
Group management	Users can form groups and online communities and manage them. The focus is on the management or administration of groups [45, 46]	Illegal content management on Zhihu, forming a “Quanzi” on Zhihu
Browsing Other's content	Users can view others' content and receive alerts to pay attention to others' content [41, 42, 45]	Browsing others' content on Zhihu, receiving notifications on LinkedIn
Meta-voicing	Users can participate in online conversations by responding to other people's status, profile, content, and activities online and viewing other people's responses to their status, profile, content, and activities. In meta-voice, the user “not only has to express his or her opinion, but also add meta-knowledge to content already online.” [23, 48]	Voting on a post on Zhihu, answering questions on Zhihu, liking what others post on Zhihu
Communication	Users can chat or send messages with others directly [41, 44, 47]	Chatting on Zhihu, communicating with others on Google+
Collaboration	Users can collaborate with others, such as collaborating with others to create content on Wikipedia [23, 43, 48]	Adding, deleting, and editing content on Zhihu
Competition	Users are able to compete with others, which includes competing in online games [10]	Completing tasks in World of Warcraft
Sourcing	Users are able to ask for resources or funds, including meeting others' requests for funds or resources [10]	Asking or answering questions on Zhihu

Knowledge affordances		
Knowledge attributes	Reliability: It refers to the extent to which the answers on social Q&A websites make users feel trustworthy and reliable [49]. Users think that the answer is of high quality only when they believe that the source and content of the answer are reliable [50]	The reliability of Zhihu content is reflected in its questions, answers, articles, videos, pictures, etc.
	Selectivity: Users can subscribe to specific content or sources of information [51]	Zhihu involves popular and unpopular content in multiple sections, and the content knowledge within each section is highly subdivided [52]
	Economies: It means that the subject obtains relatively maximum benefits with relatively minimum investment to obtain benefits most economically and meet the needs of survival and development [53]	Zhihu provides users with a free Q&A community [54]. Users can spend less money to ask questions to experts in related fields
	Uniqueness: It is defined as individuals pursuing unique characteristics different from others by acquiring, using, and disposing of consumer goods [55]. Novelty is a concept closely related to uniqueness. Novelty refers to the extent to which the answers on social Q&A websites make users feel innovative. Innovative answers will bring new ideas to users and will also be regarded as high-quality answers by users [49, 56]	In-depth content production is different from the knowledge provided by other Q&A platforms. Online and offline knowledge products are carried out at the same time [57]

**Table 4:** Mapping of Zhihu features to Zhihu affordances.

Zhihu platform features	1	2	3	4	5	6	7	8
Uploading own content	√							
Sharing other's content		√						
Watching live				√				
Commenting on other's post					√			
Asking or answering questions							√	
Liking or collecting what others posted					√			
Voting for what others posted					√			
Chatting						√		
Joining an online community			√					
Browsing other's content				√				
Following other users			√					
Searching answers							√	
Writing the column	√							
Zhihu product features								
Reliability								√
Selectivity								√
Economies								√
Uniqueness								√

1–7: self-presentation, content sharing, relationship formation, browsing others' content, meta-voicing, communication, sourcing, and knowledge attributes.

**Table 5:** Scale items.

Questionnaire 1					
Construct	Abbr.		Items		Sources

Psychological needs					
Autonomy	A		I need freely choose what I want. I need present myself in my own way I have the need to act freely		Deci [61]; Deci and Ryan [33, 62, 63]
Relatedness	R		I need participate in group activities I need connect with other people I need be close to many people		Deci and Ryan [63, 74]; Jenkins-Jenkins-Guarnieri et al. [64]; Seder and Oishi [65]
Competence	C		I need expand my knowledge I need show my capabilities I need feel competent		Bauer and McAdams [66]; White [67]
Having a place	HP		I need a place of my own I need create a place which makes me feel like home		Barki et al. [68]; Harrison and Barthel [69]; Pierce et al. [70]
Coming to know the self	CK		I need to know who I am. I need to know how other people think of me. I need to develop a sense of self-identity.		Pierce et al. [71]; Festinger [72]; Mead [73]
Expressing self-identity	ES		I need to show people who I am. I need to express my personality. I need to show my self-identity.		Pierce et al. [71]
Maintaining continuity of self-identity	MC		I need to compare my past and today. I need to show other people who I were and who I am. I need my past to be an important part of my self-identity.		Pierce et al. [71]
All items were measured on a 5-point scale: 1 = strongly disagree; 5 = strongly agree					
Questionnaire 2					
Zhihu features					

Frequency of using Zhihu platform features (aggregate of use across features)		The extent to which the use of or feeling the following Zhihu features (all items were measured on a 5-point scale Zhihu platform features: 1 = never, 5 = very often Zhihu product features: 1 = strongly disagree, 5 = strongly agree)			
		F1		Uploading own content	
		F2		Sharing other's content	
		F3		Watching live	
		F4		Commenting on other's post	
		F5		Asking or answering questions	
		F6		Liking or collecting what others posted	
		F7		Voting for what others posted	
		F8		Chatting	
		F9		Joining an online community	
		F10		Browsing other's content	
		F11		Following other users	
		F12		Searching answers	
		F13		Writing the column	
Degree of feeling Zhihu product features		F14		Reliability	
		F15		Selectivity	
		F16		Economies	
		F17		Uniqueness	

**Table 6:** Reliability and convergent validity.

Constructs	Items	Loadings	Cronbach's alpha	Composite reliability	Average variance extracted
A	A1	0.835	0.886	0.887	0.723
	A2	0.859			
	A3	0.856			

R	R1	0.738	0.748	0.752	0.503
	R2	0.698			
	R3	0.691			

C	C1	0.740	0.843	0.843	0.644
	C2	0.888			
	C3	0.771			

HP	HP1	0.801	0.767	0.767	0.623
	HP2	0.777			

CK	CK1	0.779	0.855	0.857	0.666
	CK2	0.799			
	CK3	0.868			

ES	ES1	0.724	0.801	0.808	0.584
	ES2	0.790			
	ES3	0.778			

MC	MC1	0.835	0.832	0.835	0.629
	MC2	0.780			
	MC3	0.762			

*χ* ^2^/d*f* *=* 1.330, RMSEA = 0.040, SRMR = 0.043, NFI = 0.905, CFI = 0.974, TLI = 0.967, IFI = 0.975					

Note: A = autonomy; C = competence; HP = having a place; CK = coming to know the self; MC = maintaining continuity of self-identity; ES = expressing self-identity; R = relatedness.

**Table 7:** Summary statistics and discriminant validity.

	A	R	C	HP	CK	ES	MC
A	**0.850**						
R	0.284 ^*∗∗*^	**0.709**					
C	0.325 ^*∗∗∗*^	0.560 ^*∗∗∗*^	**0.802**				
HP	0.351 ^*∗∗∗*^	0.266 ^*∗∗*^	0.311 ^*∗∗∗*^	**0.789**			
CK	0.282 ^*∗∗∗*^	0.495 ^*∗∗∗*^	0.277 ^*∗∗*^	0.393 ^*∗∗∗*^	**0.816**		
ES	0.262 ^*∗∗*^	0.559 ^*∗∗∗*^	0.483 ^*∗∗∗*^	0.268 ^*∗∗*^	0.459 ^*∗∗∗*^	**0.764**	
MC	0.256 ^*∗∗*^	0.684 ^*∗∗∗*^	0.391 ^*∗∗∗*^	0.306 ^*∗∗*^	0.654 ^*∗∗∗*^	0.446 ^*∗∗∗*^	**0.793**
Mean	4.437	3.871	4.299	4.403	3.921	3.803	3.858
S.D.	0.726	0.734	0.697	0.700	0.791	0.822	0.842

Note: ^*∗*^*p* < 0.05, ^*∗∗*^*p* < 0.01, and ^*∗∗∗*^*p* < 0.001; the diagonal elements represent the square root of the AVE.

**Table 8:** Regression results.

	SP	CS	RF	BOC	MV	COM	SO	KA
Psychological needs								
A	0.027 (0.089)	0.053 (0.097)	−0.018 (0.088)	0.309^*∗∗∗*^ (0.079)			−0.008 (0.079)	
R	0.223^*∗∗*^ (0.106)		0.245^*∗∗*^ (0.097)	−0.075 (0.084)	0.227^*∗∗*^ (0.079)	0.051 (0.130)		
C					0.270^*∗∗∗*^ (0.078)		0.366^*∗∗∗*^ (0.084)	0.369^*∗∗∗*^ (0.055)
HP	0.300^*∗∗∗*^ (0.092)	0.260^*∗∗∗*^ (0.101)						
CK				0.246^*∗∗*^ (0.078)	0.189^*∗∗*^ (0.066)			
ES	0.219^*∗∗*^ (0.084)	0.179^*∗*^ (0.088)	0.340^*∗∗∗*^ (0.085)		0.167^*∗*^ (0.067)	0.114 (0.116)		
MC	0.013 (0.088)	0.092 (0.087)						
Controls								
Gender	0.079 (0.123)	−0.021 (0.134)	−0.106 (0.125)	−0.095 (0.111)	0.031 (0.095)	0.050 (0.171)	−0.009 (0.113)	0.052 (0.077)
Age	0.047 (0.089)	−0.035 (0.097)	0.142^*∗*^ (0.090)	−0.004 (0.080)	−0.044 (0.069)	−0.085 (0.122)	−0.126 (0.080)	−0.056 (0.055)
IE	−0.102 (0.122)	−0.046 (0.132)	0.019 (0.125)	0.065 (0.110)	−0.101 (0.093)	−0.075 (0.169)	0.092 (0.108)	-0.117 (0.073)
*R* ^2^	0.321	0.180	0.274	0.193	0.417	0.043	0.170	0.161
Adj *R*^2^	0.294	0.151	0.252	0.169	0.397	0.019	0.149	0144

Note: ^*∗*^*p* < 0.05, ^*∗∗*^*p* < 0.01, ^*∗∗∗*^*p* < 0.001, standardized coefficients (standard errors); IE=Internet experience; Gender: 1 = male, 2 = female; SP = self-presentation; CS = content sharing; RF = relationship formation; BOC = browsing others' content; MV = meta-voicing; COM = communication; SO = sourcing; KA = knowledge attributes.

## Data Availability

The data are available from the corresponding author upon request.
